# Freeride Skiing—Risk-Taking, Recognition, and Moral Boundaries

**DOI:** 10.3389/fspor.2021.650564

**Published:** 2021-03-11

**Authors:** Gustav Tøstesen, Tommy Langseth

**Affiliations:** ^1^Department of Sport, Food and Natural Sciences, Faculty of Humanities, Sports and Educational Sciences, Western Norway University of Applied Sciences, Sogndal, Norway; ^2^Department of Sports, Physical Education and Outdoor Studies, Faculty of Humanities, Sports and Educational Sciences, University College of Southeast Norway, Telemark, Norway

**Keywords:** risk-taking, freeride skiing, cred-zone model, extreme sports, social recognition, Pierre Bourdieu, moral boundaries, Michele Lamont

## Abstract

Freeride skiing is an activity that is, or at least can be, quite dangerous. Risk-taking in high-risk sports has usually been understood within a psychological framework. Building on Pierre Bourdieu's sociology, this article highlights the social dimension of risk-taking in freeride skiing by scrutinizing values within a freeride culture. A central question in this article is: what kind of actions are given recognition and credibility in freeride skiing? The findings show that there is a clear link between risk-taking and credibility and that risk-taking might be seen as a form of capital. However, risk-taking's link to recognition is not straightforward—it is limited by the skiers' skill level. To further develop our understanding of the social dimension of risk-taking we use Michelle Lamont's theory of symbolic boundaries. By expanding the Bourdieusian understanding of social practice with Lamont's work, we gain insight into how risk-taking is socially regulated by social conventions within a subculture. This means that we in this article describe three social dimensions of risk-taking: (1) The link between risk-taking and recognition, (2) The limits of the risk-recognition nexus, and (3) The moral boundaries of risk-taking.

## Introduction

Sunny and nice weather, untouched terrain, fresh powder. Maybe some cliffs to jump off. That is every freeride skier's dream. But with this dream comes risk. Risk of avalanches, falling off cliffs, sliding uncontrolled down a steep mountainside. Despite the risk, freeriding—skiing outside the boundaries of resorts and in the mountains—has become ever more popular, both internationally (Frühauf et al., 2020) and in Norway, the focus of this study (Bischoff and Odden, 2000; Odden, 2008; Evju, 2014). The rise in freeride ski participation can be seen as part of a general increase in interest in extreme/high risk-sports. Langseth (2011) suggests that the increase in high-risk sport participation can be understood in two ways: Either as an adaptation to late-modern cultural norms such as individuality and creativity, or as a form of compensation in a society where life is experienced as boring and dull and where the individual has little influence over their own life. Echoing the latter, from a psychological perspective, Frühauf et al. (2019) state that participants in high-risk sports lack a feeling of agency in their everyday lives, but that this can be found in such sports. In a comparison between freeride skiers and “slope skiers,” Frühauf et al. found that freeride skiers had experienced higher levels of agency than slope skiers before and after riding. They also scored higher on sensation-seeking. This is maybe unsurprising, since freeride skiing involves more objective danger than skiing the slopes of a resort (Haegeli et al., 2012; Frühauf et al., 2019, 2020; Niedermeier et al., 2020). It would be one-dimensional to see risk-taking and sensation-seeking as the only factors that make people take up freeride skiing. Still, this article will focus on risk-taking. Not necessarily to say that it is the most important explanation of why people participate in freeride skiing and other high-risk sports, but because the mechanisms behind risk-taking are still little understood.

### Risk-Taking, Sport, and Society—Some Brief Notes

Risk is a multifaceted concept. We can for instance talk about economic risk, social risk, and physical risk (Donnelly, 2004). Even though both economic and social risk could be part of being highly involved in a sport subculture, the most prominent form of risk when it comes to sport is physical risk. Voluntary risk-taking involving physical risk has been investigated from various research perspectives. Within psychology, risk-taking is seen as dependent on one's innate personality type (e.g., Zuckerman, 1979, 2007; Farley, 1986; Breivik, 1996). Personality type simply makes some people high-sensation-seekers and others low-sensations-seekers. Within this framework, then, freeride skiers are most likely high-sensation-seekers. Other theorists, most notably Csikszentmihalyi, focus on the “thrills” and “flow”–the good feelings that freeride skiing and other high-risk sports entail (Csikszentmihalyi, 1991; Jackson and Csikszentmihalyi, 1999).

However, these individualistic approaches to risk-taking, do little to explain the general growth of interest in high-risk sports. Macro-oriented sociologists on the other hand, has tried to explain risk by pointing toward changes in modernity. Wagner (1994) states that a key feature in the rise of the welfare states in the late nineteenth century was the rationalization and collectivization of risk. Nevertheless, as Beck (1992) has argued, modernity started to produce its own risks. The expansion of science and technology created unpredicted risks and a move toward what Beck names “risk society” (Beck, 1992). A hallmark of risk society is extensive reflexivity toward risks and the “*potentially disastrous effects of modernisation*” (Giulianotti, 2009, p. 550). Stranger (2007) holds that voluntary risk taking can be a way for participants in risk sports to show themselves that they can handle living in a risk-society. In a metasociological review of how sociologists with a macro focus tries to explain the growth of risk-sports, Langseth (2011) found two major strands: a compensation perspective and an adaptation perspective. The compensation perspective holds that we are living in a society that is too safe and too regulated. Participation in risk sports can be seen as a way of compensating for the social constrains the individual agent is embedded in. Elias and Dunning's *Quest for Excitement* (Elias and Dunning, 1986), although dealing with sports in general, not only risk sports, can be seen as typical for this perspective. As affections and emotions have become increasingly supressed throughout modernity, human drives and desires becomes ever more disciplined (Elias, 2000). This creates and unrelenting tension between inner drives and socially demanded drive-control. In sports, Elias and Dunning (1986) argues, these norms are temporarily dissolved. The quest for excitement in sports can thereby be understood as a modern variation of the “raw” desire found in earlier societies. Within the compensation perspective, then, participating in risk sports is seen as a way of creating a balance between inner, “true,” drives, and the external, modern constraints that are placed on each individual. The adaptation perspective, on the other hand, holds almost the opposite view: Participation in risk sports is not about distancing from or escaping modernity, but rather an adaptation to modernity's cultural imperatives such as flexibility, individualism, and anti-conventionalism (e.g., Crosset and Beal, 1997; Palmer, 2004; Arnegård, 2006).

As shown, agents' motivations for voluntary risk-taking have been widely studied both from psychologically, individualistic micro perspectives and sociological macro perspectives. As we see it, the micro perspectives tends to overlook cultural dynamics and the macro perspectives on the other hand have a tendency to overlook individual emotions and affections. With the concept “edgework” Lyng (1990, 2005) tries to bridge micro and macro by utilizing a combination of Marxian and Meadian analysis. While emphasizing the positive experiences of edgework, Lyng acknowledges, in line with the compensation perspective, that risk-taking can be seen as reactions toward social alienation (Lyng, 1990). He also recognizes that edgework might be an “*expression of the central institutional and cultural imperatives of the merging social order*” (Lyng, 2005, p. 878). In other words, the emotions and affections involved in edgework is connected to macrosociological structures and cultural values. In this article, we maintain that the motivation for participation in risky activities is highly social, but our focus is on how the individual is influenced by group level processes. To put it another way, our study holds a meso perspective. That is not to say that experiences of “flow” and “thrill,” or changes in modernity are not important when it comes to understanding risk-taking practices. Rather, our aim is to bracket the meso-level in order to detail how risk-logics develops within a specific sport subculture.

Several studies have emphasized how specific contexts shapes risk rationalities within sports (Hunt, 1995; Booth, 2003; Donnelly, 2004; Laurendeau, 2006, 2008; Beedie, 2007; Fletcher, 2008; Langseth, 2012a, 2013, 2016). Based on the insight from these articles, this article explores the relational aspect of risk taking. Bunn has in several works used the theoretical framework of Bourdieu to investigate how climbers through socialization within the climbing culture develops a habitus that makes them pre-reflexively prone to the risk assessment needed to climb certain routes (Bunn, 2016, 2017a,b). While also building on Bourdieu, our article takes another approach. It follows a line of thought from Booth (2004) and Booth and Thorpe (2007) where it is argued that prestige and recognition within risk sports is connected to risk taking. Further, it builds upon Langseth's studies on how risk-taking can be seen as a form of symbolic capital that gives BASE jumpers (Langseth, 2012b, 2016) and rock climbers (Langseth and Salvesen, 2018) recognition and credibility within their milieus. Langseth and Salvesen developed a “cred-zone model.” This model states that to gain recognition for a high-risk act, the athlete must have the right balance between level of risk and skill. In this study, we have studied how these mechanisms work within the freeride culture in Norway. Our first research question is, then, *What value does risk-taking have in the freeride skiing community in Norway?* To study this, we have used Bourdieu's sociocultural perspectives and conceptual apparatus. This means that our research object is not individual traits. Our research object is relational; it is the social, group-level, sense-making processes, the processes that makes risk-taking rational and even logical, that interest us. In other words, it is not individual motivation or experiences that we are studying, but rather how values at a group level influence the actors' decision making.

Langseth and Salvesen (2018) found that even though risk-taking could be seen as a form of symbolic capital, risk-taking had its limits. When the athlete was not seen as being skilful enough for the amount of risk involved in an act, the act was deemed foolhardy and did not confer any credibility. In this article, we will dig deeper into the line between what is seen as cred-worthy and what is seen as foolhardy. We do this by using Michelle Lamont's understanding of symbolic boundaries (Lamont, 1992). So, our second research question is: *How are symbolic boundaries constructed in connection to risk-taking within the freeride ski culture?* By applying Lamont's boundary approach, we wish to achieve two things. First, we want to get a deeper understanding of the social mechanisms behind risk-taking in high-risk sports. Lamont's perspective will help us understand what regulates and keeps risk-taking in the freeride culture at bay. Second, we want to expand Bourdieu's theory of practice by highlighting that there are moral and symbolic boundaries to the accumulation of symbolic capital.

## Materials and Methods

To gain insight into the social aspects of risk-taking in freeride skiing, this study relies on qualitative interviews with seven informants (four men, three women). Before we go deeper into methodological issues, it is important to also say that both authors have been, if not core members, at least avid freeride skiers with personal connections at the core of the Norwegian freeride culture. That means that we had a good understanding of the values of the freeride culture before we started the research. One could ask the question of whether this means that we had preconceived notions about the ski culture. That might be, but as this is a heavily theoretical, informed reading of our empirical data, we would suggest that our own initial understanding of the research data is not problematic. We will come back to that later.

The seven informants recruited for this study all lived in a small town on the west coast of Norway that we have chosen to call “Westfjord.” Because of its proximity to alpine terrain, Westfjord has become a hub for freeride skiing in Norway. Since it has a university, it attracts a lot of students who are already into skiing. It also attracts mountain guides looking for work. All the informants have freeride skiing as their main interest. None of them were beginner freeride skiers, but their experience level still varied. Some had been active freeride skiers for more than 10 years, whereas others were relatively new to freeriding. Their ages varied from 18 to 45 years. It is important to note that even if some of them were new to freeriding they had all been skiing all of their lives. The informants were recruited by one of the authors through Messenger. This way the informants could see that the researcher also lived in Westfjord and that he was not a complete outsider to the ski community. The idea was that it would be easier for the informant to say yes to being part of the study. The informants were all skiers that we knew by reputation, but we did not know them personally.

To gain insight into the skiers' own narratives of risk-taking, we used what Kvale and Brinkmann (2009) call semi-structured interviews. The strength of semi-structured interviews is that the researcher can follow an interview guide containing the issues that she or he wants to cover, but at the same time be flexible enough to follow up on interesting themes that come up during the interview (Kvale and Brinkmann, 2009). The interviews lasted between 60 and 85 min. They were all recorded and transcribed in their entirety. This process was carried out in accordance with the guidelines for ethics in the social sciences in order to protect the dignity, rights, and welfare of the research participants. All participants received written information about informed consent before agreeing to be interviewed. The transcribed material was anonymized and safely stored at closed database after the interview process.

The analyses of the transcribed material were done by using the conceptual frameworks of Bourdieu and Lamont. This means that we do not advocate an epistemology of continuity. As Bourdieu argues (Bourdieu and Wacquant, 1992), social scientists should not be satisfied with just re-presenting informants' stories. Instead, these stories should be re-interpreted in the light of theoretical concepts. This means that the actors in the freeride ski culture and we as researchers probably have very different understandings of the logic behind their skiing practices. Several theorists (Hellevik, 1999; Kvale and Brinkmann, 2009; Bryman, 2012) argue that it is impossible to believe in complete objectivity; however, it can be argued that Bourdieu's theoretical and methodological position can help maintain a critical distance from the material and a particular awareness of the origin and perspective of the material (Bourdieu and Wacquant, 1992; Wilken, 2008). In the context of a research project, it is always necessary to question and break with the established truths that exist within the culture under study in order to reconstruct this as a research object within a theoretical framework (Bourdieu et al., 1991). From this perspective, it is important to problematize and objectify the answers from the informants, as they represent both a socially constructed set of values, and a socially constructed way of talking about these values (Bourdieu et al., 1991). Bourdieu's theory tries to generate concepts that can capture how actions are conditioned by the social system of which the actor is a part (Wilken, 2008). Values and meanings do not carry any pre-existing meaning beyond their social context (Wilken, 2008). With this in mind, it is possible through analytic interpretation to both see the stories from the informants as real events that carry significant personal meaning to the practitioners, and at the same time critically deconstruct the narratives and understand them as values and meanings developed within a certain (sub)cultural framework.

## Theoretical Approach

One way of breaking with the informants' mundane understanding of practice, is, according to Bourdieu (1999, 2004), to understand practice through the lens of the theoretical concepts. In the present study, we will use the concept of *capital* to gain knowledge of risk-taking within the freeride ski community in Westfjord. Before we move on, we have to give a short description of Bourdieu's theoretical framework.

### Symbolic Capital and Field

Capital, In Bourdieu's sense (Bourdieu, 1999), exists in three basic forms: cultural capital, economic capital and social capital. In addition, there exists a fourth type of capital in which the three basic types can appear, namely symbolic capital (Wilken, 2008). When a capital form is assigned a symbolic value, it appears as a significant capital form. For example, a pair of skis (economic capital) may appear as a status symbol (symbolic capital) (Wilken, 2008). Bourdieu argues that symbolic capital can appear in a variety of forms (Bourdieu, 1999). In our case, we use the term symbolic capital to study the specific values within the freeride ski community.

A central point in Bourdieu's theory is that recognition is a fundamental social force. The driving force behind all investments in a social field is the pursuit of recognition (Bourdieu, 1999). Recognition is the foundation of this field's capital system and especially what Bourdieu calls symbolic capital. Symbolic capital, he writes: “*(…) exists (…) only in and through the reputation, recognition, faith, trust and reputation of other people, and it can no longer be preserved until […] it manages to maintain the belief that it exists”* (Bourdieu, 1999, p. 173). In other words, symbolic capital can only be considered a currency when it is recognized by the agents in the field in question. Following Bourdieu's theory, then, possessing symbolic capital in the form of valid skills and knowledge will define whether a skier achieves social recognition.

Bourdieu's concept of *field* is an analytical tool used to make sense of different forms of struggles over capital within specific social systems. Field refers to relations between agents that compete about specific forms of capital (Wilken, 2008, p. 40). A skier's position in the ski-field is dependent upon his or her relative amount of field-specific capital. Within the field, capital is understood as a practitioner's resources and skills that provide both an opportunity to exercise power within the ski culture and to gain recognition and status. The community and the social context created by the skiing activity can in this view be seen as a dynamic “social game.” Practitioners will constantly position themselves against each other by producing and reproducing dominant standards in the freeride ski community. It is central to Bourdieu that the rules of the game are not rigid and static but that they are forever negotiated. In this view, practitioners in the field will position themselves relative to each other based on their amount or volume of capital. Skiers can increase status and recognition by increasing their capital but also by changing the relative value of capital. For Bourdieu, the driving force in the field becomes the power struggle between the different positions (Bourdieu, 1999). It is central to Bourdieu that the agents do not necessarily have a conscious understanding that they are taking part in power struggles within a social field. Thus, freeride skiers do not consciously know that they are part of this social game.

### The Credibility-Zone Model

**Figure 1:**
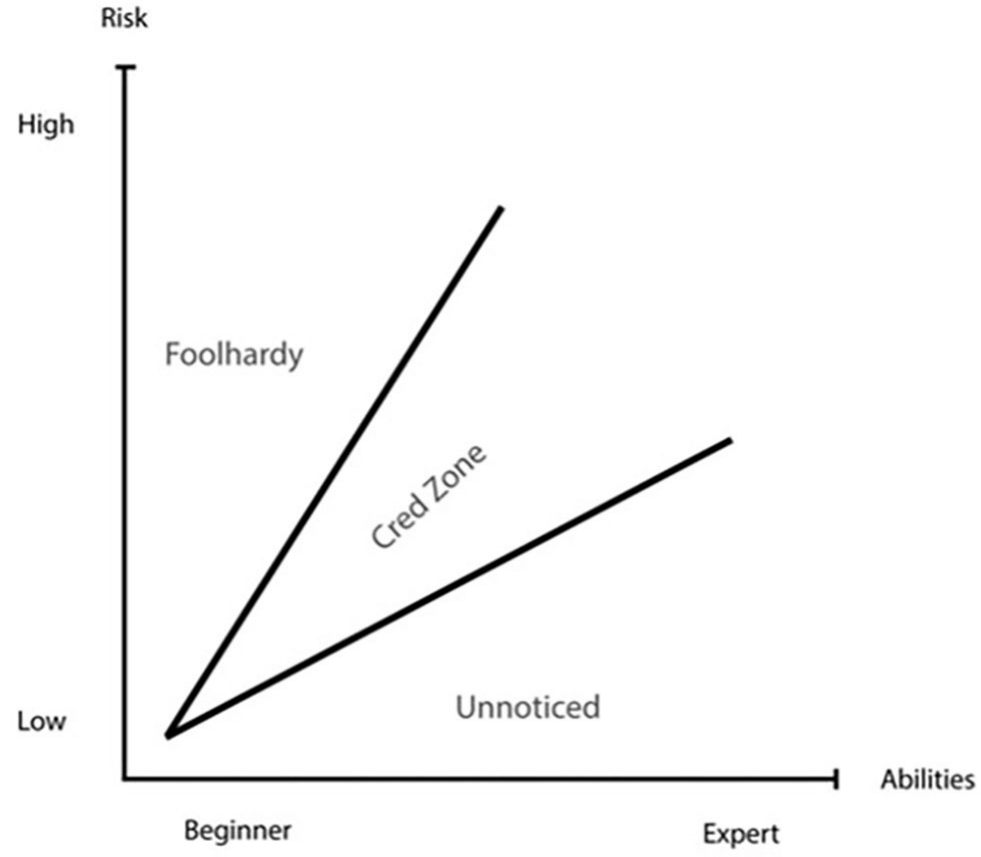
The credibility-zone model from Langseth and Salvesen (2018).

Based on a Bourdieusian framework, Langseth and Salvesen developed a *Credibility-Zone model* to study risk-taking in rock climbing (Langseth and Salvesen, 2018). In their study, Langseth and Salvesen (2018) found that in rock climbing, risk-taking can be seen as a form of symbolic capital. However, they also found that there are limits to risk-taking as capital: if a climber takes too much risk, the action does not give recognition but is rather seen as foolhardy. The cred-zone model was developed to make sense of this logic and tries to capture the relationship between skills, risk, and recognition. It has a risk on its x-axis and skills on the y-axis (Figure 1). The findings of Langseth and Salvesen (2018) indicate that in the middle of the model, the “cred zone” emerges. To gain recognition, the practitioners have to have the right balance between risk-taking and skills (Langseth and Salvesen, 2018). A beginner would be deemed foolhardy for taking the same risks for which an experienced climber would be recognized. On the other hand, an experienced climber climbing an easy, safe route would not receive recognition—the climb would go unnoticed.

In this study, this model is built upon as a tool to investigate the distribution of recognition in the freeride ski community. The cred model will serve as a starting point for investigating risk-taking within the freeride ski culture in Westfjord.

## Results and Discussion

As mentioned earlier, voluntary risk-taking is most commonly understood in psychological terms, where biology, intrinsic motivation, and emotions are the main explanatory factors. Conversely, the social dimensions leading to risk-taking have been studied to a lesser extent. A narrative of individualism, which seems to disguise the social dimensions and make it difficult to get informants to talk about risk-taking in other that individualistic terms, surrounds the ethos of action sports. This might have to do with the late-modern “ethics of authenticity,” as Taylor (1991) called it, that makes interviewees prone to giving answers that are individualistic, connected to intrinsic motivation or emotions. Although a quick glance at freeride skiing on social media platforms would tell us otherwise, participants seem unwilling to talk about the social dimensions of risk-taking. “The best skier is the one with the biggest smile” is a quote often heard among skiers. But is that the truth? Would a happy beginner be considered the best skier? Probably not. In freeride skiing, as in any other sport, there are values that skiers adhere to and that make skiers “smile” when they achieve them. These values are not individual but are developed relationally within the subculture of freeride skiing. Following Bourdieusian thinking, we could say that intrinsic motivation is not “intrinsic,” at least not in a naïve understanding of the term, but rather extrinsic as it stems from a social field. In Langseth's (2012b; 2016) study of BASE jumpers, and Langseth and Salvesen's (2018) study of climbers, voluntary risk-taking was found to be a central value and could be seen as a form of symbolic capital that leads to recognition within those communities. The question here, then, is whether risk-taking can be seen as a value in freeride skiing. If so, how and under what circumstances can it lead to prestige and recognition?

### The First Dimension of Risk: Risk-Taking Equals Recognition

Before we turn to the freeride community in Westfjord, let us first take a brief look at some structures that most likely affect how freeride skiers view risk-taking: freeride ski competitions and ski movies. In freeride skiing competitions, participants ski down a steep, exposed, and often dangerous mountainside. It is not the time that determines the winner, but the skiers “style” and line choice. In general, the more difficult the line, the more points awarded. And, usually, a more difficult line means a more exposed and dangerous line. In other words, in freeride skiing competitions, the connection between risk and recognition is institutionalized. Now, freeride competitions are not the same as freeriding in general, but their system of valuation might influence freeride skiing in its everyday form. Another influence might be ski movies. Ski movies, with their focus on steep descents, jumping off big cliffs etc., establish narratives that connect risk to recognition.

Several of the informants from the ski community made references to ski movies that inspired them. The influence from movies is clear when our informant “Mie,” herself a professional freeride skier, says:

“In competitions, you are always told to ski like they do in the movies. That's the standard. That's what everybody is reaching for. If you are good enough or not is seen up against it [what they do in movies]. And here in Westfjord there are many skiers that are as good as in the movies and they are seen as tougher than the others…”

Clearly, movies and the athletes that appear in them have some kind of definitory power when it comes to value in the freeride ski culture. Being a key figure in the freeride culture on a national level, “Elias” touches upon this theme when he says that: “*Ski movies play a role in defining what is cool. You know, it is often extreme ski films that are published*. *They clearly represent some form of ideal. But you have to have a conscious attitude toward that*.” This quote reveals both that ski movies are influential and that Elias thinks that skiers should be a bit critical toward the content. This latter point will be addressed further in a section below, but for now, it is the definition of common values in the community and how risk-taking becomes a value in itself that are central.

Freeride competitions and ski movies are one thing; everyday reality might be another. The question is, then, do the same mechanisms—the connection between risk and recognition—also exist in settings that are more mundane? To get a sense of the hierarchies, and thereby the values, of the freeride ski culture in Westfjord, we simply asked our informants about who they saw as “good” skiers and why. In others words, what kind of skiing is given recognition? When “Mie” was asked what good skiing is, she responded: “*What looks nice (…), and then you need to land jumps from cliffs and ski continuously. It should also be a bit steep.”* So, besides the fact that the skiing should look aesthetically pleasing, it is also important that it contains jumps or drops from cliffs and that it happens in steep terrain. In other words, she connects risk and recognition, as she clearly means that “good skiing” involves risk. However, all the informants mention that there is a big difference between skiing steep and doing huge drops near resorts and doing the same things in the mountains. The freerider and “steep-skier” “Håvard” describes that he thinks it is really impressive when skiers take their skills from the skiing resorts into the big mountains: “*I think it is impressive if somebody sends big drops and sticks them in the mountains. (…) Like wow, 10 meters, scmack! Without doubting.”* The quote reveals that big mountain skiing has high status. It is also an example of the connection between risk and recognition. The same actions performed near a resort do not give the same credibility. This means that it is not just the actions or the skills in themselves that give status. The increased risk of being in big mountains, far from help if anything should happen, gives the actions more value. Further, the mountain guide “Even” says: “*If you absolutely want to be a part of the steep skiing milieu, then you must be aware that you have to take the necessary risks.”* In Bourdieu's language, we could say that risk-taking in itself becomes a form of capital that gives the skier an opportunity to increase her or his status and thereby climb the hierarchy of the freeride community. Another mountain guide and professional skier “Kjetil” tells us that the riders who are considered the best, the skiers at the top of the hierarchy, are there at least partly because they have the form of capital that is connected to risk-taking:

“The skilled skiers ohhh they are in a league of their own. It is clear that if you are going to get cred (…) of course you have to do some things that are on the edge. If you are going into that community, then there is no point in just skiing ordinary things.”

To us it seems clear that for freeriders in Westfjord risk and recognition are connected. This is in line with values in other high-risk sports such as BASE jumping and rock climbing (Langseth, 2012b, 2016; Langseth and Salvesen, 2018). Risk-taking can be seen as a form of symbolic capital within the freeride community in Westfjord and maybe also in the freeride field in general. It can give the holder of that specific capital an opportunity to climb in the social hierarchy of freeride skiing. Risk-taking as capital belongs to what we would like to call the first dimension of risk-taking. However, this is a form of capital that is socially regulated: there are social mechanisms at work that regulate this kind of market.

### The Second Dimension of Risk: Recognition Is Limited by Skills

Talking about how Instagram is used in Westfjord, “Mie” says:

“Here in Westfjord, it is extreme how all (skiing) terrain becomes visible through Instagram. The freeride milieu is pretty small and everything becomes very visible, especially where the good riders have been. Then this is pursued by skiers at a lower level who should not be there and might not know what kind of terrain they are in, both when it comes to their own skills and avalanches.”

Two important processes are going on in the quote above. First, it is clear that what the “good” skiers are doing is dangerous and that is a part of what they are recognized for. Secondly, the quote reveals that gaining recognition for doing dangerous things is limited by skills. As seen in the quote, “Mie” warns against beginners trying to ski the same lines as highly skilled riders. That probably means that riders who are lacking skills would not get recognition for doing the same line as the acknowledged riders. This resembles what Langseth and Salvesen (2018) found in the cred model, that there is a significant logic or balance between risk and abilities. So, taking risk does not in itself automatically entail recognition: to gain recognition, the skier should both have bodily skills to ski well and also skills to judge the avalanche danger where they are skiing. The latter requires a few extra comments. The risk of avalanche is not the kind of risk that entails recognition. Knowingly skiing in an area where there is a high chance of being caught by an avalanche would not give even the most skilled skier recognition. However, the lines that are steep enough to confer recognition are also within the same terrain steep enough for avalanches to appear. So, there seems to be a line that is constructed between riders who know enough about avalanches and those who don't. How those lines are drawn is hard to tell; however, it is clear that the same action at the same place is not given the same value when the skier is not seen as being proficient.

This logic is seen when “Håvard” talks about how he meets “fresh” students every year in Westfjord who try to become core members of the freeride community:

*“I think it is important that the community focus on the fact that people are skilled, make good decisions and let people hear it if they don't. Every year, somebody that doesn't have a clue tries to become something. They make mistakes in the mountains, they are loose cannons. You don't ask those to come along*.”

As discussed above, it is clear that in order for a new student to enter the social hierarchy of freeride skiing, risk-capital is essential. But again, the quote also shows that there are limits to risk-taking as a form of capital. If you are considered a “loose cannon” it is likely that performing an action that in other cases would have conferred cred is discredited. To pinpoint exactly where the line between actions that give cred and actions that are deemed foolhardy sits is not easy. These are blurred boundaries. The freeride community, as with most lifestyle sports, is a community with a heavy individualistic ideology that probably makes it hard for the athletes to come up with clear-cut, concrete statements about these boundaries. Competition skier “Ida” says “*It is easy to think that you have to let them do what they want to and think for themselves what they are capable of. After all, it is not my job to determine their skill level*.” Still, this is something that is discussed between the core members of the subculture. As mentioned by Langseth and Salvesen (2018), when Canadian skier Jamie Pierre in 2006 set a world record for highest drop when he jumped of an 82-m cliff, many discussions started online at the Norwegian ski magazine *Fri Flyt*. Some commentators thought it was awesome, while others saw it as plain stupid. The reason for this disparity was that Pierre didn't do the drop in a controlled manner; he basically landed on his head, but still got out of it unhurt. Another interesting case when it comes to the cred/foolhardy boundary is the discussions after Killian Jornet, a Spanish sky runner and ski mountaineer currently living in Norway, in 2018 skied down a 1,600-m climbing route close to the infamous Troll Wall. The event was widely discussed within the Norwegian ski community and we asked our informants what they thought about it. “Mie” said: “*He does things on a completely different scale than everyone else (…) his risk assessment is completely different from ours. I do not think he takes a high risk (…) because (others) are not as good as Killian*.” Here we see a clear contrast to the new, striving, students in Westfjord that are seen as “loose cannons.” We see that as long as a skier is seen as highly skilled, she or he is given cred for actions that otherwise would have been seen as foolhardy.

In a similar manner, “Håvard” describes the “good” freeride skier in a general way: “*The one who can ski everything and make it in a controlled manner, skiing steep, skiing at high speed (…) and be able to choose good lines.”* Clearly, in Håvard's view, being a “good” freerider is connected to competence and experience along with having control in different kinds of situations. Furthermore, Håvard describes that the “good” skier not only possesses these qualities but is also willing to take a calculated risk: *"It often has something to do with risk management. That they have made a good assessment of their skills and risk (…) so they end up with a good result.”* Here Håvard highlights a theme that is common to all the informants and follows the logic of the cred model, namely that risk-taking is legitimized by high competence and experience. This means, as previously mentioned, that the symbolic capital connected to risk-taking is regulated by others' thoughts about a rider's skill. However, what these skills are, what these boundaries consist of, remains unclear.

### The Third Dimension of Risk: Moral Limits to Risk-Taking

Even if these boundaries are blurred, it is still clear that some actions involving risk give credibility while others are deemed foolhardy and still others are just passed in silence. But what kind of boundaries are these? In Bourdieu's understanding there would not be any boundaries to capital accumulation. Reading too many books wouldn't stop you from increasing your cultural capital. To get a better understanding of these boundaries, it is necessary to move beyond a purely Bourdieusian scheme. When skiers discredit some acts because they are seen as foolhardy, we would say that these are acts of moral judgement that limit the accumulation of the type of symbolic capital connected to risk. According to Lamont (1992), moral boundaries are among the blind spots of Bourdieu's theory. Following Lamont, symbolic boundaries are the lines that people draw when they categorize people and when they make distinctions between who they associate with and those they resist associating with. We combine this perspective with Bourdieu's to get a sense of the boundaries that skiers draw between acts that are given recognition and acts that are seen as foolhardy, even if it is the same type of symbolic capital that is utilized.

“Ida” touches upon the morality of risk-taking when she talks about how some athletes at freeride ski competitions push themselves above their abilities:

“You see them fall and roll and roll. And lying still when they stop (…) it is incredibly disgusting (…) It affects not only oneself but everyone who loves them. And it's incredibly uncomfortable and it puts so many others at risk.”

Putting others at risk seems to be part of the moral boundary. She points toward how others (families and friends) are or should be part of the considerations for a freeride athlete. In other words, the freeride skier has moral obligations that supersede the risk-recognition logic. This indicates that morality is an important factor in the distribution of recognition. Still, the morality of risk-taking is less tangible than the risk-recognition logic. But it is clear that we are in a moral landscape. “Håvard” talks about how it is necessary to take responsibility but that “*There are some who do not manage to take that responsibility (…) and they will make a fool out of themselves.”* The words “responsibility” and “fool” are words that set these statements within an understanding of practice that has to do with morals. The use of the word “responsibility” also shows that this is not just an individual endeavor and points toward the social limits of risk-taking. A few years back, there was an accident in Westfjord. In this accident, a male skier was taken in an avalanche while skiing a steep line. He was buried in the snow debris for hours before the alpine rescue group found him. He had skied alone and had not told anyone where he went. In the interview guide, we used this accident as a case that might reveal feelings and values about risk-taking among the informants. “Kjetil” described his feelings about the accident:

“It's clear that he (…) made a fool out of himself (…). It just had to go wrong (…). It's personal decisions. I do not blame him for what he did. It's his own life. (…) This is a person who is damn good at skiing, likes to take chances “living on the edge” (…). He dropped a cliff and landed on a minefield that took him.”

Again, we see that “fool” is used to describe the accident. And a “fool” is a word that is used to describe people who do not adhere to moral norms. The same tendency is seen in the descriptions from the local skier “Lisa:” “*The stupidest thing was that he went alone and did not inform anyone about the trip destination. Maybe he was super good at* (reading) *avalanches and maybe he was super good at skiing, but you never know (…) it was stupid*.” As an outdoor teacher, “Lisa” holds the skier's actions up against common norms of outdoor leisure in Norway: you should not travel alone in the mountains and you should always tell others where you go. The fact that the skier did not follow these common rules and norms of behavior resulted in a moral distance between him and other skiers. Six of all seven participating informants reacted as “Lisa” did with negative moral associations when talking about the skier involved in the accident. The informants also linked the skier's actions to moral considerations about rescue personnel or close relatives. As a part of the rescue group, “Kjetil” revealed ambiguous feelings about the accident:

“It was crazy to start a rescue operation on grade 3 in a valley that is a large terrain trap. That's why I reacted. We had no control over the situation. It was (…) not good.”

Here he describes how the rescue in his opinion was dangerous because the valley was not safe (avalanche level 3 in the Norwegian system) and it was in his mind not rational to start the operation. The same understanding is seen when the mountain guide “Even” talks about the accident:

“People have to decide and make decisions about their own lives, but at some point it will affect others (…) family and such, also with rescue personnel. I am open for a discussion about how far one can go. (…) I want to live.”

As described earlier, the moral boundaries in relation to this skier are created on the basis of a moral judgement about common responsibilities and obligations to others. The fact that the skier was seen as putting other people at risk and the way relatives probably were emotionally affected afterwards created significant feelings and a certain ambivalence among the informants. This indicates a limit to recognition and what is seen as acceptable in the skiing community. According to Lamont (1992), moral boundaries create identity by producing group identities. It is “us,” the responsible freeride skiers, vs. “them,” the irresponsible “fools” and “loose cannons.” This resembles the relationship between established and outsider groups described by Elias and Scotson: “*Stigmatization as an aspect of an established-outsider relationship is often associated with a specific type of collective fantasy evolved by the established group. It reflects and, at the same time, justifies the aversion-the prejudice-its members feel toward those of the outsider group”* (Elias and Scotson, 1994, p. xxxiv). However, in our case, if the persons that stigmatizes other skiers as fools, belongs to an established group with privileges and whether “them” is a real or imagined group of skiers, or if it is just used to describe skiers that have been unlucky, is hard to tell from our interviews. From our limited empirical material, it seems that the out-group is both an imagined group of people and connected to specific situations and accidents. Laurendeau (2008) has described the process of “blaming the victim” after skydiving accidents. The same process seems to be going on here. If the skier had not been caught in the avalanche, he might just as well have been given recognition for skiing the same line. But, blaming the victim, as we see it, is connected to a significant moral content and moral boundaries. As Laurendeau and Moroz (2012) has shown, newspapers often morally condemns “backcountry adventurers” that have been victims of accidents. Our data shows that this *moral blaming* of the victim is not just something that is constructed from outside the culture, but also internally. As we have displayed above, in an accident, the discussion of what went wrong is transformed into a moral discussion on whether the skier in question had the right skills and therefore if she or he *should* have been skiing that specific line.

Another facet of the morality of risk-taking becomes evident when one of informants talked about an accident he was involved in while working as a guide:

“I think you will be judged. In positive or negative ways, but I think you will be judged. I have the impression that if you have been close to something, then you can… you should of course wish it had not happened. There is a lot…eeh… some kind of shame is probably involved in it.”

The use of the word “shame” is interesting. Since he was working as a guide when this happened, it has some different connotations than if it was just him taking risk. But it still points in the direction that taking too much risk is connected to shame. Shame is a complex social phenomenon, but it is always connected to moral norms in one way or another. In Langseth and Salvesen's (2018) presentation of the cred model, the optimal relationship is located between experience and risk and the right capital composition. However, Lamont's theoretical perspective on moral boundaries shows how this optimal relationship is expressed and practiced as a moral boundary. While the analyses show that Bourdieu's concepts of field and capital are useful when it comes to understanding the mechanisms behind risk-taking, we would also suggest that Bourdieu's theory should be expanded by including moral boundaries. According to Lamont (1992, p. 11), at a macro sociological level, boundary work is used to reinstate order within communities by reinforcing collective norms. In terms of freeride skiing we can see these moral boundaries as a way to ensure that there are not too many accidents. A risk sport only functioning according to Bourdieusian mechanisms of capital accumulation would probably swiftly go off the rails and see too many accidents. Moral boundaries serve as a way for the freeride ski culture to self-regulate.

## Conclusion—Three Dimensions of Risk-Taking

The main objective of this study has been to acquire an understanding of the mechanisms behind risk-taking in freeride skiing. Based on our analysis of freeride skiers in “Westfjord,” we found that there are three central dimensions that influence risk-taking.

*The first dimension* establishes a relation between risk-taking and recognition. Often, the motivation for risk-taking is understood as individual ambitions. For some it is understood as genetic propensities that makes certain people take extended risks. Others see this as motivated by the “thrills” and “flow” that such actions entail. We do not say that these understandings are false, but our findings show that they are not the whole story. Building on a Bourdieusian framework, we show that risk-taking can be seen as a form of symbolic capital that can give the holder recognition and status. Risk-taking is thereby a social phenomenon. What the agents strive for, what they dream about doing, and what they actually do on their skis does then stem from being socialized into a subculture that holds certain values. The central point is that risk-taking is a value in freeride skiing. That means that the motivation for taking risks cannot be seen as “intrinsic.” The motivation stems from incorporating the values held in the freeride community. In other words, these values are from the outset extrinsic, but as skiers are socialized into this community the values become intrinsic. This creates a logic that is deeply embedded among freeride skiers and connects risk-taking and recognition.

However, *the second dimension* of risk-taking shows that this logic has its limits. Building on Langseth and Salvesen's Cred-Zone model (Langseth and Salvesen, 2018), we show that the risk-recognition logic is limited by the riders' skill levels. Taking risks does not automatically entail recognition. For a rider to be in the “Cred Zone,” the risk they take has to be in accordance with their skill level. A beginner taking too much risk would be deemed foolhardy and would not get recognition for the act. Likewise, a highly skilled skier skiing in easy terrain would go unnoticed and this would not help her or him to gain status within the subculture of freeride skiing.

The limits of the risk-recognition logic are further explored in *the third dimension* of risk-taking. Here, we explore risk-taking and recognition by employing Michelle Lamont's concept of *moral boundaries* (Lamont, 1992). Failing to ski within your limits is connected to skills. But it is not the skills *per se* that are evaluated, but rather that not having the right amount of skill makes a skier break some moral boundaries. The freeride skier has moral obligations that go beyond the individual athlete—the skier should, according to the informants, take friends, family, and rescue personnel into the equation when they undertake risky skiing. Failing to do so could lead to both a personal feeling of shame and criticism from the freeride community. We propose that these moral boundaries can be seen as social mechanisms that work to regulate the risk-recognition logic in the freeride community. These collective norms, we think, keep the number of accidents down. But at the same time we could also speculate that these moral boundaries and the feelings of shame that could be felt if these norms are broken could also make it hard to talk about and learn from accidents in hindsight.

The contributions of this article are three-fold. *First*, we wanted to highlight the complexity of voluntary risk-taking in extreme sports from a sociological understanding of group-level processes. *Second*, the article shows both the usefulness of a Bourdieusian framework for understanding social actions but also the need for an expansion of Bourdieu's understanding of social practice. Our analysis shows that the accumulation of symbolic capital is limited by a moral dimension—a moral dimension that cannot be reduced to a form of symbolic capital, but that belongs to another dimension of sociality that is not well-described by Bourdieu himself. *Thirdly*, we hope that the three dimensions we propose will be used in future research in other research projects investigating risk-taking in sports and other activities.

## Data Availability Statement

The raw data supporting the conclusions of this article will be made available by the authors, without undue reservation.

## Ethics Statement

The studies involving human participants were reviewed and approved by NSD. The patients/participants provided their written informed consent to participate in this study.

## Author Contributions

All authors listed have made a substantial, direct and intellectual contribution to the work, and approved it for publication.

## Conflict of Interest

The authors declare that the research was conducted in the absence of any commercial or financial relationships that could be construed as a potential conflict of interest.
